# Abundance and Stability as Common Properties of Allergens

**DOI:** 10.3389/falgy.2021.769728

**Published:** 2021-10-28

**Authors:** Alexander C. Y. Foo, Geoffrey A. Mueller

**Affiliations:** Genome Integrity and Structural Biology Laboratory, National Institute of Environmental Health Sciences, Durham, NC, United States

**Keywords:** allergens, stability, abundance, exposome, allergenicity

## Abstract

There have been many attempts to identify common biophysical properties which differentiate allergens from their non-immunogenic counterparts. This review will focus on recent studies which examine two such factors: abundance and stability. Anecdotal accounts have speculated that the elevated abundance of potential allergens would increase the likelihood of human exposure and thus the probability of sensitization. Similarly, the stability of potential allergens dictates its ability to remain a viable immunogen during the transfer from the source to humans. This stability could also increase the resilience of potential allergens to both gastric and endosomal degradation, further skewing the immune system toward allergy. Statistical analyses confirm both abundance and stability as common properties of allergens, while epidemiological surveys show a correlation between exposure levels (abundance) and allergic disease. Additional studies show that changes in protein stability can predictably alter gastric/endosomal processing and immunogenicity, providing a mechanistic link between stability and allergenicity. However, notable exceptions exist to both hypotheses which highlight the multifaceted nature of immunological sensitization, and further inform our understanding of some of these other factors and their contribution to allergic disease.

## Introduction

Allergic diseases, characterized by a Th2 immune response to typically innocuous environmental antigens, are one of the most common health disorders worldwide with a prevalence of 1–20% depending on the region (1). Respiratory allergies are often comorbid with asthma, hypertension, and other respiratory disorders while food, drug, and venom allergies can result in life-threatening anaphylactic reactions in up to 5% of the American population, highlighting the burden of allergic disease on human health and wellness (1–4). Despite the size of the exposome, it has been repeatedly noticed that allergens which are capable of eliciting a Th2 allergic response are generally distributed across relatively few protein families, suggesting the presence of specific biophysical properties which promote sensitization (5). Understanding the factors which differentiate allergens and non-allergens can provide valuable insight into the mechanism of allergic sensitization, with potential implications for the design of novel immunotherapies and mitigation strategies.

Allergic sensitization is a multifaceted process, a comprehensive discussion of which is beyond the scope of a single work. Instead, this review will focus on recent work examining two frequently-cited factors: allergen stability and abundance. Intuitively, the link between abundance, stability, and allergenicity appears straightforward with higher abundance correlating with an increased risk of exposure. Similarly, higher stability increases the length of time allergens will persist in the environment, further increasing the likelihood of exposure. However, these seemingly obvious concepts are surprisingly controversial. For example, many studies have attempted to correlate allergen exposure levels with sensitization. However, there remain uncertainties concerning the most relevant routes of exposure, and dominant reservoirs of allergenic material (6). Similarly, many longitudinal studies have failed to demonstrate predictable dose response relationships (6). Likewise, a systematic investigation into the role of stability in allergen exposure is an equally daunting endeavor with variable definitions of protein stability. For instance, aeroallergens must endure dehydration, oxidative exposure, and UV radiation, while in the context of food science one must consider the effects of gastric digestion and food preparation. Some fields may eschew both definitions altogether and focus instead on a more biophysical approach centered on thermal/chemical denaturation or conformational stability. Further complicating the conversation is the need to consider the role of stability and abundance in multiple contexts. For example, recent work suggests that food allergy to peanuts is the result of co-exposure to the skin and intestine where different measures of exposure and stability may apply (7).

It is worth reminding the reader that exposure to potential allergens is only the first step in the sensitization process. From here, potential allergens must be processed, and the resulting antigenic fragments presented to the immune system for recognition. The intensity, duration, and timing of this presentation are also important factors (8, 9). The prevalence of allergens and their resistance to both gastric and endosomal proteolysis could alter the kinetics of this process, providing an additional mechanism through which stability and abundance influence allergenicity while offering insights into the sensitization pathway (10, 11). This review will highlight recent literature examining the role of antigen stability and abundance in the sensitization process, along with several notable exceptions to this trend. The biochemical and biophysical mechanisms which mediate this interaction will be examined, along with their implications for human health and disease.

## Abundance and Stability as Important Characteristics of Allergens

The correlation between abundance and allergenicity is intuitively easy to understand, but significantly harder to verify in a rigorous manner. Circumstantial evidence for such a relationship can be found in the observation that food allergens such as Pen a 1 (shrimp), and Ara h 1 and Ara h 2 (peanut) comprise a significant portion (up to 20%) of the total protein that humans consume when eating shrimp and peanuts (12–14). Similar results have been reported for both indoor and outdoor aeroallergen sources such as cockroach frass (Bla g 1, 18% total protein), Timothy grass pollen (Phl p 5, 6.4% total protein), and house dust mite extracts (Der p 1, ~33% total protein) (15–17). Epidemiological studies provide further evidence for an empirical link between abundance and allergenicity. For example, comparing the levels of cockroach allergen in house dust samples from households with cockroach-allergic and non-allergic children reveals a strong linear correlation between sensitization and allergen (Bla g 1 and Bla g 2) content, with some studies showing a sensitization rate upwards of 80% in households with the highest exposure levels (18, 19). Similar results have been reported for dust-mite allergen exposure and sensitization, though no dose-response relationships for other indoor allergens such as mold, dog, or cat were identified (20–22). The methodological challenges associated with assessing environmental allergen exposure could in part contribute to these inconsistent results. For instance, airborne allergens are extremely prevalent in the community, and can be found in public spaces such as schools, offices, and daycares at levels sufficient to promote sensitization independent of household exposure (23–26). Additionally, the presence of allergic symptoms or a familial predisposition to indoor allergies could encourage households to actively reduce exposure through avoidance or mitigation strategies, yielding a spurious inverse dose-response correlation among some study subjects (27). Finally, most environmental exposure studies fail to report the prevalence of allergens relative to their non-allergic counterparts. One should note that the motivation of these studies was to understand the environmental factors which contribute to allergenicity; a goal in which they have succeeded admirably. However, this limitation hinders the efforts of our present review to assess abundance as a biophysical determinant of allergenicity in an empirical manner.

Two recent studies have addressed this major research gap in the field. These studies used RNA-Seq to quantify the transcription levels of 39 allergens and >1,000 non-allergenic proteins from cockroaches, dust mites, and tree, weed, and grass pollens; providing an empirical measurement of abundance within their respective allergen sources (28, 29). While the abundance of potential allergens contributes to the likelihood of human exposure, the ability to resist environmental degradation also plays a key role. In recognition of this, the authors employed chemical denaturants coupled with high-throughput mass-spectrometry techniques to simultaneously assess the biophysical stability of their protein samples. The large sample sizes employed in these studies allowed the authors to demonstrate with a high degree of statistical certainty that allergens were generally both more stable and more abundant than their non-allergic counterparts. Similar analyses have been carried out on food allergens, though given their route of exposure a greater emphasis has been placed on their ability to resist gastric digestion (30–32). While less extensive than their aeroallergen counterparts, these works do suggest that food allergens are indeed more resilient than their non-allergenic counterparts.

It should be noted that the global stability of a protein may not reflect that of its immunogenic fragments. For instance, the peanut allergen Ara h 3 is broken down when subjected to simulated gastric digestion (33). However, IgE-reactive regions remain intact, allowing it to retain its sensitizing potential (33). Likewise, the peanut allergens Ara h 2 and Ara h 6 survive digestion, and remarkably can be detected in breast milk, remaining immunologically intact after lactation (34, 35). A different process was noted for the allergens Ber e 1 (Brazil nut), Jug r 2 (Walnut), and Ara h 1 (Peanut). *In situ* proteolytic cleavage results in the release of a small vicilin-buried peptide (or peptides), which possess their own immunogenic activity independent of the vicilin allergen (36–38). Even with this consideration of immunogenic fragmentation, the correlation between exposure, and allergenicity is far from complete. A closer examination of some of these exceptions provides invaluable insight into the sensitization process.

## Anomalous Dose-Response Curves Provide Insight into the Mechanisms of Allergic Sensitization

**Figure 1:**
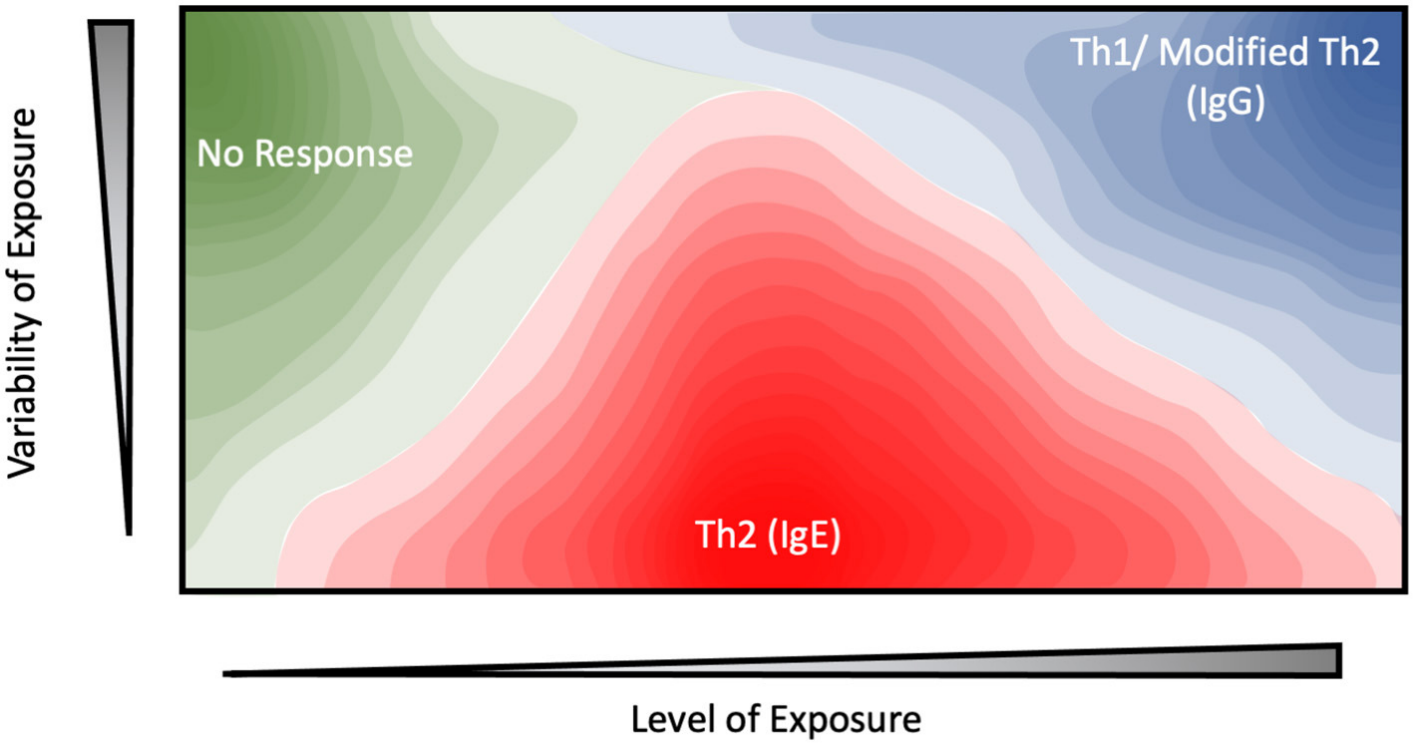
Contour plot depicting the proposed relationship between the level of exposure, variability of exposure, and immune response for potential environmental allergens.

While abundance generally correlates with allergenicity, other factors such as the timing or duration of exposure also need to be considered. Exposure to occupational allergens is often consistent and well-defined, providing a viable platform for exploring this perspective. Indeed, a study examining the correlation between mouse allergy and occupational exposure among animal facility workers revealed a parabolic relationship, with moderate (1 ng/m^3^) levels of atmospheric Mus m 1 resulting in a 10-fold increase in the odds ratio for sensitization, while exposure levels either above or below this level reduced this risk to near-baseline levels (39). Interestingly, the authors observed that the day-to-day variability of exposure levels played an equal, if not more important role with consistent, low to moderate dosages being required for allergic sensitization while high variability and high-dosage regimes resulted in a reduction in both serum IgE and allergic symptoms. This dovetails well with studies examining household sensitization, in which exposure to mouse allergen levels below this 1 ng/m^3^ threshold yield a linear dose-response curve (40). Another study examining occupational allergies among University personnel in Brazil yielded similar results. Here, the concentration or intensity of mouse or rat allergen within the work environment was not a significant risk factor for sensitization (41). Instead, the duration of exposure and job category (e.g., technician vs. student) emerged as major determinants of allergenicity. While the role of variability was not specifically examined in these studies, the results are consistent with the model shown in Figure 1 in which the time-course of presentation plays an equal, if not more important role than mouse allergen concentration or exposure intensity.

Similar results have been observed in beekeepers, whose occupational exposure to the bee allergen Api m 1 is highly intermittent (42, 43). Under this highly-variable exposure regime, allergic sensitization and serum IgE levels were inversely correlated with average dosage level, as measured by the number of stings received. In one paper, ~80% of keepers with the lowest exposure levels (<25 stings per year) displayed sensitization against Api m 1 (44). This figure drops drastically to 35% for keepers who received 25–200 stings per year, while no systemic response was observed at all among those which received the highest dosages (>200 stings a year) (42–45). These results suggest a model in which high-intensity and variability exposure regime elicit a protective effect, while moderate, sustained dosages are more likely to generate an allergic response (Figure 1).

A few comments on the mechanisms of allergic tolerance are in order before returning to the central theme of abundance and exposure. In the studies mentioned above, tolerance to the bee venom Api m 1 was driven by the conversion of both Th1 and Th2 cells into IL-10 secreting Tr1 cells, giving rise to what has been termed a modified Th2 response (44, 46–48). In this model, IL-4 induces B-cell activation and antibody production, similar to the standard Th2 response. However, the simultaneous presence of IL-10 stimulates B-cell class-switching from IgE antibody production to IgG_4_, while depletion of Th2 cells also reduces the secretion of IL-4 and other pro-inflammatory cytokines (49–51). While some Th1-Tr1 conversion was observed, the authors noted that Tr1 class switching was mediated primarily through histamine receptor 2 (HR2) (48). HR2 acts primarily to suppress Th2 cell proliferation and cytokine secretion. Like its Th1-associated subclasses (IgG_1−3_), IgG_4_ is generally viewed as a protective antibody which has been shown to block IgE binding and cross-linking against a range of allergens (46, 52), though some studies challenge its protective effects (53). Nonetheless, both IL-10 production and IgG_4_ class-switching remain strongly associated with a reduction in serum IgE and allergic symptoms, giving rise to the protective effect observed among beekeepers (48). Similar class-switching has been observed in laboratory workers in mice and rat facilities, suggesting that this modified Th2 response contributes to allergic tolerance in a wide range of environmental allergens (39, 52, 54). Here, allergic tolerance was correlated to both IgG_4_ and IgG_1−3_. While the latter was not found to be statistically significant, it is consistent with other studies in which the protective effect of immunotherapy treatments is mediated by a plurality of IgG isoforms suggesting both the classic Th1 and modified Th2 response can contribute to tolerance (55–58). Despite its protective effects, the modified Th2/IgG_4_ response is dependent on initial B-cell activation by IL-4. As such, the kinetics of IL-10 expression are critical to avoid terminal Th2 differentiation, with *in-vitro* experiments suggesting a 72 h narrow window in which IgG_4_ class-switching must occur (59). Additionally, IL-10 production by dendritic cells is extremely transient, being detected only within 24–72 h of antigen exposure (60). A frequent, high-intensity exposure regime potentially increases the probability that the kinetics of IL-10 production coincides with the narrow window required for modified Th2 response and IgG_4_ class-switching, promoting allergic tolerance against occupational allergens under these conditions.

While occupational allergen exposure levels and timing can be easily defined, the same generally cannot be said for household allergens, hindering efforts to determine whether the latter follow the same dose-response model as shown in Figure 1. One interesting approach to overcome this challenge is embodied by the work of Woodcock et al. in which they actively altered the household environment through the use of allergen mitigation strategies, and assessed their effect on allergic sensitization and serum IgE levels (61). These interventions include the use of allergen-impermeable bed covers, vinyl flooring materials and a rigorous laundry schedule among others, and were effective at significantly reducing the levels of Der p 1, Fel d 1 (cat) and Can f 1 (dog) allergens ~2 to 3-fold relative to a control group. The treatment group displayed a significant increase in serum IgE against Der p 1, but not Fel d 1 or Can f 1 despite their airborne allergen levels being suppressed to the same degree. One potential explanation stems from the differences in exposure patterns. In a household setting, mite and cockroach allergens are found within relatively large particles (>10 μm). Due to their size, the majority of mite allergens remain confined to their reservoirs until physically disturbed (62, 63). In practice, this results in relatively low exposure to ambient airborne mite allergens, punctuated by transient periods of high exposure as the resting reservoirs are disturbed. For example, time-based measurements of personal mite allergen exposure indicate that initial entry into the bed yielded an intense >100-fold increase in airborne Der p 1 concentrations (64). However, allergen levels quickly returned to their resting-phase concentrations within 20 minutes following disruption (47, 63), ensuring that the sleep phase accounted for a small minority (~10%) of total exposure despite the bed being a major allergen reservoir (23, 24). Similarly, agitation of carpets and other allergen reservoirs during daytime activity such as walking or bedmaking results in the resuspension of a large number of allergen-containing particles, which rapidly settle down and dissipate in under an hour (65, 66). The intervention levels introduced by Woodcock et al. eliminate these transient spikes, potentially contributing to the increased sensitization. In contrast a greater proportion of dog and cat allergens are carried by smaller (<5 μm) particles which remain airborne for significantly longer (2–14 days) following disruption, resulting in a much more consistent exposure pattern regardless of human intervention (64, 67, 68). An important assumption in these studies is that the majority of the allergen exposure occurs in the home. This has been challenged by studies using personal air samplers (24), and studies of allergen prevalence in schools (69). Despite these many concerns, it is notable that Fel d 1, Can f 1, and Der p 1 share similar non-linear dose-response curves, with home exposures above 20 μg of allergen per gram of house dust being associated with reduced allergenicity and an increased IgG response (70–73). This suggests that the model described in Figure 1 can be broadly applied to both workplace and environmental allergens.

It has been consistently observed that the prevalence of both inhalation and food allergies is significantly lower in rural populations when compared to their urban counterparts, providing an additional avenue through which the effect of household environment on allergic sensitization can be examined (74–76). As with the Woodcock study, a significant reduction in mite sensitization and serum IgE was observed among children living on farms despite experiencing higher exposure levels within their homes than their more urban counterparts (77). One potential explanation would be that the former spend significant portions of their day outside their homes, resulting in a higher variability in mite allergen exposure as they transition from the outdoor to indoor environment. This is in contrast to the latter which spend the majority of their waking hours indoors where they receive a consistent, if lower intensity dosage of mite allergens. This inverse correlation between indoor and animal allergen exposure and sensitization among rural and agricultural communities has been widely reported (78, 79), and provides a valuable tool for dissecting the role of allergen exposure in sensitization. However, it is important to remember that other environmental differences could also contribute to this urban/rural divide. For instance, a comparison of Amish and Hutterite farming communities points to endotoxin exposure as a key determinant of sensitization: while both populations share numerous genetic and cultural similarities, the more traditional agricultural practices of the former result in significantly higher exposure to levels of airborne bacterial endotoxins and lower levels of indoor allergen sensitization (80, 81). The higher prevalence of endotoxin found in rural/farm dwellings relative to their urban counterparts could play a similar role (78). While a comprehensive assessment of the environmental determinants of allergy is beyond the scope of this review, suffice it to say that allergic sensitization is a multifaceted process in which the abundance of the potential antigen and the timing of exposure are important, but often not the sole contributors.

## Antigen Presentation Kinetics and Stability Influence Allergic Sensitization

**Figure 2:**
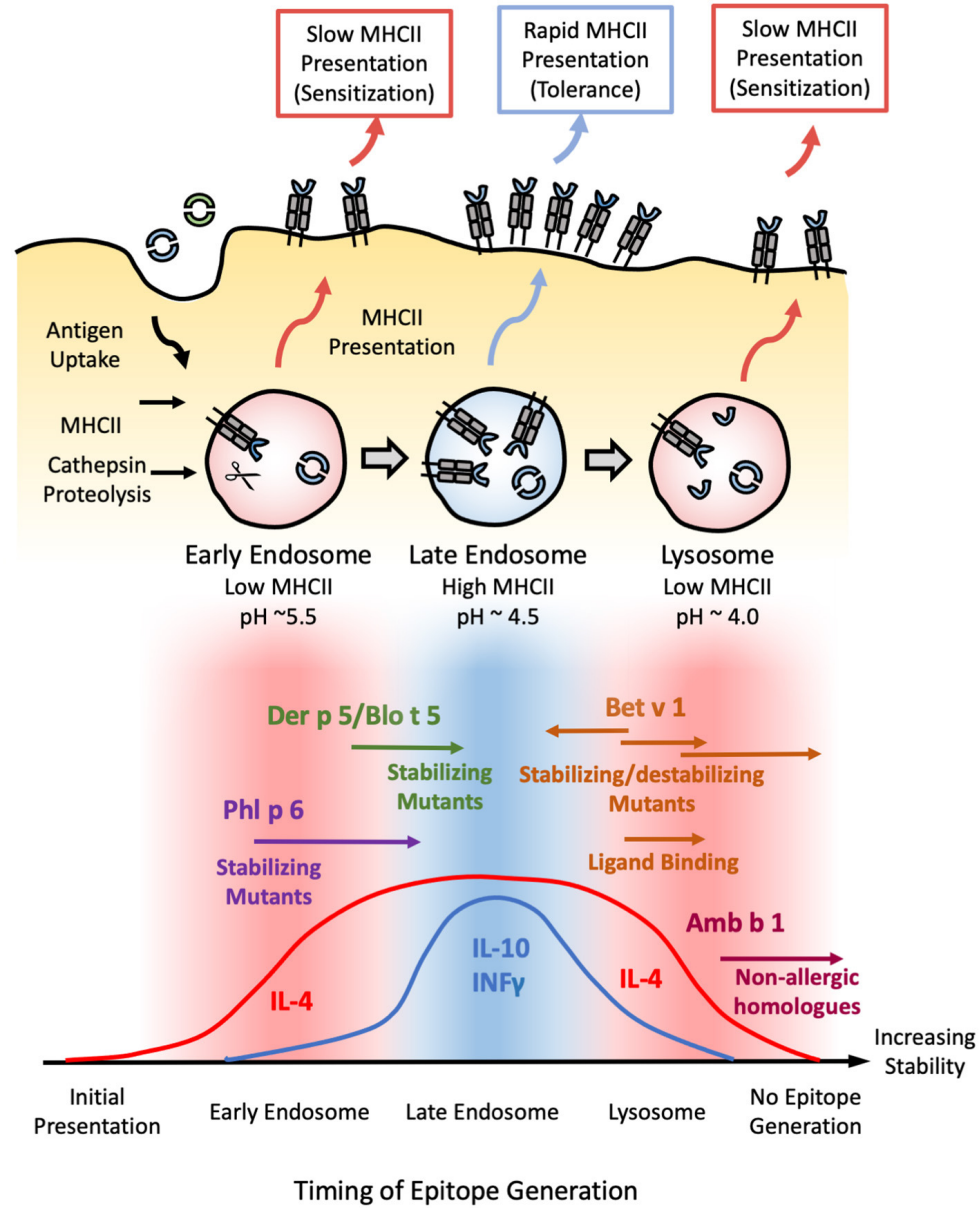
Model correlating allergen stability against endosomal degradation and immunogenicity. **(Top)** Schematic illustrating the process of endosomal degradation and epitope presentation. Antigens are internalized into the endosomes of antigen presenting cells, where they are subjected to cathepsin proteolysis under reducing and increasingly acidic conditions. The resulting epitope fragments are loaded onto MHCII molecules for presentation and T-cell recognition. MHCII loading is most efficient in the late endosome. As such, epitopes generated in this stage are more likely to generate a protective Th1 or modified Th2 response defined by the presence of both IL-4/IL-10 and/or INFγ (tolerance). Conversely, epitopes generated in the early endosome or lysosome are more likely to yield an IL-4-dominated Th2 response (sensitization) due to the scarcity of MHCII in these compartments, resulting in a more gradual, sustained, yet low intensity mode of presentation. **(Bottom)** Based on this model, exposure to proteins with an intermediate stability against endosomal degradation are more likely to induce tolerance (blue), while proteins which deviate from this medium in either direction are more liable to develop allergic sensitization (red). This is illustrated with Phl p 6 and Der p 5/Blo t 5; and Bet v 1 and Amb a 1 as representative high and low-stability allergens respectively. Stabilizing mutants shift the former into the intermediate-stability regime, resulting in a tolerogenic response. In contrast, enhancing the stability of the latter via mutations, chemical modification, or ligand binding enhances their sensitizing potential, while destabilizing mutants of Bet v 1 have the opposite effect. Note that antigens which occupy either extreme of this scale are expected to elicit no immune response due to the lack of epitope generation and/or MHCII loading, as illustrated by the low immunogenicity of extremely stabilized variants of Amb b 1 or Bet v 1.

A mechanistic basis for this unusual dose-response relationship may be attributed to the underlying process of T-cell activation. To be recognized as an allergen, exogenous antigens must first be internalized into the endosome of an antigen presenting cell (APC) such as the dendritic cells discussed above. Internalized antigens are then subjected to endosomal degradation, where they are exposed to cathepsin proteases (Figure 2) under increasingly acidic and reducing conditions. The resulting peptide fragments are loaded onto the class two major histocompatibility complex (MHCII) and presented on the cell surface for recognition by T-cell receptors (TCR). The kinetics of MHCII-antigen presentation and TCR binding can influence T-cell differentiation. High, but transient concentrations of MHCII-peptide complexes stimulate the production of tolerogenic cytokines and suppression of IL-4 consistent with a Th1 or modified Th2 response. Conversely, lower, but more persistent presentation skews the immune system toward an IL-4-dominated Th2 response (8, 82, 83). As one might expect, the abundance and exposure regime of an allergen directly contributes to the kinetics and intensity of MHCII presentation, with large, acute exposures resulting in allergic tolerance as shown in beekeepers and mouse facility workers as discussed in the previous section (84). However, the biophysical stability of an allergen and its ability to resist endosomal degradation can also influence MHCII-presentation kinetics. This was examined using the major birch allergen Bet v 1. Bet v 1 was among the least stable proteins tested by Cabrera et al. (29). However, this observation fails to consider the fact that Bet v 1 in its natural allergen source exists as a complex mixture of different isoforms, each with different biophysical properties (85). For example, Bet v 1a (Bet v 1.0101) is significantly more stable, with noticeably reduced conformational flexibility than Bet v 1d (1.0102) as assessed using solution-NMR (86). The increased stability of the former increases its resilience to Cathepsin S proteolysis, resulting in slow MHCII loading and associated Th2 polarization, while its less stable counterpart is associated with a protective IgG response (85, 87, 88). Similarly, nitration of Bet v 1—mimicking the effect of air pollution in industrialized nations—resulted in the formation of higher-order oligomers. The resulting complexes displayed enhanced resistance to endosomal degradation, and were more liable to generate a Th2 response than their unmodified counterparts (89). This trend is not only limited to Bet v 1. For instance, loss of conserved disulfide bonds severely compromised protein structure and stability of Pru p 3 (peach) and Art v 1 (Mugwort), reducing allergenicity (90, 91). A more systematic approach is presented by Ohkuri et al. Here, they created a series of Phl p 7 and hen egg lysozyme (HEL) variants with varying degrees of biophysical stability, and quantitatively assessed their ability to elicit a protective IgG response. Their results reveal a surprisingly strong inverse linear correlation between the ΔG of unfolding and IgG production (92). Applying a linear extrapolation to their data yields a ΔG value of ~22 kcal/mol, above which allergic sensitization (i.e., no protective IgG response) can be expected (92). The folding energy of globular proteins tend to lie in the ~5–20 kcal/mol range indicating that allergens tend to be more stable than their non-allergenic counterparts (93, 94).

The majority of MHCII loading occurs at the late endosome (pH~4.5–5.5), while early endosome (pH ~6) and terminal lysosomes (pH ~4.0–4.5) are poor in MHC II molecules (95), suggesting that the pH dependence of fold stability must also be considered. This is illustrated by a study carried out by Machado et al. examining the immunogenicity of various stabilized Bet v 1 mutants. The mutant which generated the strongest IgE response was found to be extremely stable at pH <5, but was susceptible to limited proteolysis under the more acidic conditions found within the late endosome and terminal endosome (96). Mutants which inhibited proteolysis under all conditions were markedly less immunogenic, suggesting an upper limit beyond which further enhancements reduce immunogenicity through suppressing MHCII presentation (96). Such a conjecture is supported by subsequent studies on the pectate lyase C family, in which non-allergic members were found to be significantly more resistant to endosomal proteolysis than Amb a 1: the major allergen from ragweed (97). These results allow us to build a model in which proteins with intermediate stability elicit a protective IgG response, with the majority of their antigenic fragments being released in the late endosomes for rapid MHCII loading and presentation. In contrast, proteins which are able to survive into the lysosome before succumbing to proteolysis stimulate an allergic IgE response due to the scarcity of MHCII molecules, while extremely stabilized proteins fail to yield any appreciable MHCII loading even after prolonged digestion (Figure 2).

From this conjecture, one could extrapolate that proteins which deviate in the other direction (i.e., less stable) can also trigger a Th2 response due to the scarcity of MHCII molecules in the early endosome, where the majority of antigenic fragments are produced. This could explain the allergenicity of Phl p 6 and Der p 5—neither of which displayed a significant enhancement in biophysical stability relative to their non-allergenic counterparts (29). Stabilizing the Phl p 6-fold resulted in a shift toward a Th1/modified Th2 response, while destabilizing mutants had the opposite effect (11). Similarly, a stabilized N-terminal truncated form of the Der p 5 homolog Blo t 5 reduced IgE production without altering the repertoire of T-cell epitopes generated (98), confirming that both allergen families lie in the “low stability” island of Th2 polarization. Stabilizing/destabilizing mutants have been shown to elicit similar effects in both dust mite Der p 2 and hen egg lysozyme (99, 100). Thus, while fold stability is a major determinant of allergenicity, pH stability must be carefully considered within the context of the endosomal degradation process.

MHCII is synthesized with an invariant chain (Ii) which is cleaved upon maturation, leaving a short “placeholder” peptide (CLIP) bound into the MHCII epitope pocket. In order to be loaded onto MHCII, prospective epitopes must first displace the bound CLIP peptide: a process which is aided by the accessory molecules HLA-DM (DM) and HLA-DO (DO), which catalyzes the peptide exchange process (101). Thus, the ability of prospective epitopes to bind DO/DM and displace CLIP play a key role in immunogenicity (102–104). This is illustrated in a recent study in which the CLIP sequence from antigen presenting cells was exchanged for antigenic sequences from Bet v 1, Art v 1, and Cry j 2 (Japanese cedar). This significantly enhances the apparent affinity of these peptides for MHCII, greatly boosting antigen presentation and T-cell activation by these recombinant APC's (105, 106). Likewise, conjugating the cat allergen Fel d 1 to the Ii sequence significantly enhanced MHCII presentation, promoting a modified Th2/IgG_4_ response and allergen tolerance in human subjects during phase I/IIa clinical trials (107). Similarly, studies examining the therapeutic potential of dendritic cells modified to endogenously express mite allergens showed skewing toward a Th1 and modified Th2 response, though the specific mechanism through which this protective effect was induced was not directly assessed (108). Conversely, the presence of other DM/DO/MHCII ligands in the endosomal milieu could competitively inhibit MHCII loading (106). Such an interaction might account for the increased prevalence of mite sensitization among patients who are co-exposed to other allergens, though this conjecture has yet to be demonstrated on the molecular level (109). Further complicating this model is the observation that binding of antigenic fragments to DM/DO and MHCII shields them from further proteolysis (102–104). Thus, while the model shown in Figure 2 provides a biophysical basis for the role of protein stability in MHCII loading and allergenicity, there are always other factors to consider.

Even with this expanded understanding it is important to remember that exceptions will always exist. For instance, a survey of tropomyosin allergens from both food (Pen m 1, Shrimp; Ani s 3, fish parasite) and environmental (Der p 10, dust mite; Bla g 7, cockroach) sources revealed significant variation in both thermodynamic stability and endosomal degradation kinetics (110). However, neither measure of stability was found to correlate with allergenicity. A separate survey of 15 allergens and non-allergens found that resistance to gastric, but not endosomal degradation predicted allergenicity (111). However, a pairwise comparison of LTP, albumin, tropomyosin, collagen, and parvalbumin allergens with their non-allergic or weakly allergic homologs indicated that resistance to gastric digestion was not a strong predictor of sensitization potential (112). Taken together, these contradictory findings suggest the presence of additional factors which may complement or, in some cases supersede the influence of protein stability in the sensitization process—some of which will be discussed in the following sections.

## Lipid Ligands as Modulators of Abundance and Stability

Previous studies have shown that many allergen families are capable of binding lipids and other hydrophobic ligands (113). This activity could allow allergens to perturb biological barriers or deliver immunomodulatory ligands such as bacterial lipids or pollen-associated lipid mediators directly to the immune system, facilitating sensitization (114–118). However, the presence of lipid ligands or adjuvants could influence both abundance and stability, further contributing to allergenicity. For example, lipid emulsion significantly enhanced the resistance of allergenic fish parvalbumins to gastric digestion, facilitating exposure of the intact antigen to the immune system (119). Similarly, mustard (Sin a 2) and peanut (Ara h 1) allergens have been shown to interact on the surface of phospholipid vesicles derived from their respective allergen source, protecting them from gastric digestion and increasing the apparent abundance of intact antigen upon encountering intestinal immune cells (120). This interaction also impaired antigen uptake and endosomal degradation of both Ara h 1 and Sin a 2, altering the kinetics of MHCII presentation and ultimately skewing the immune system toward a Th2 response in a manner similar to the stabilized Bet v 1 mutants (120).

**Figure 3:**
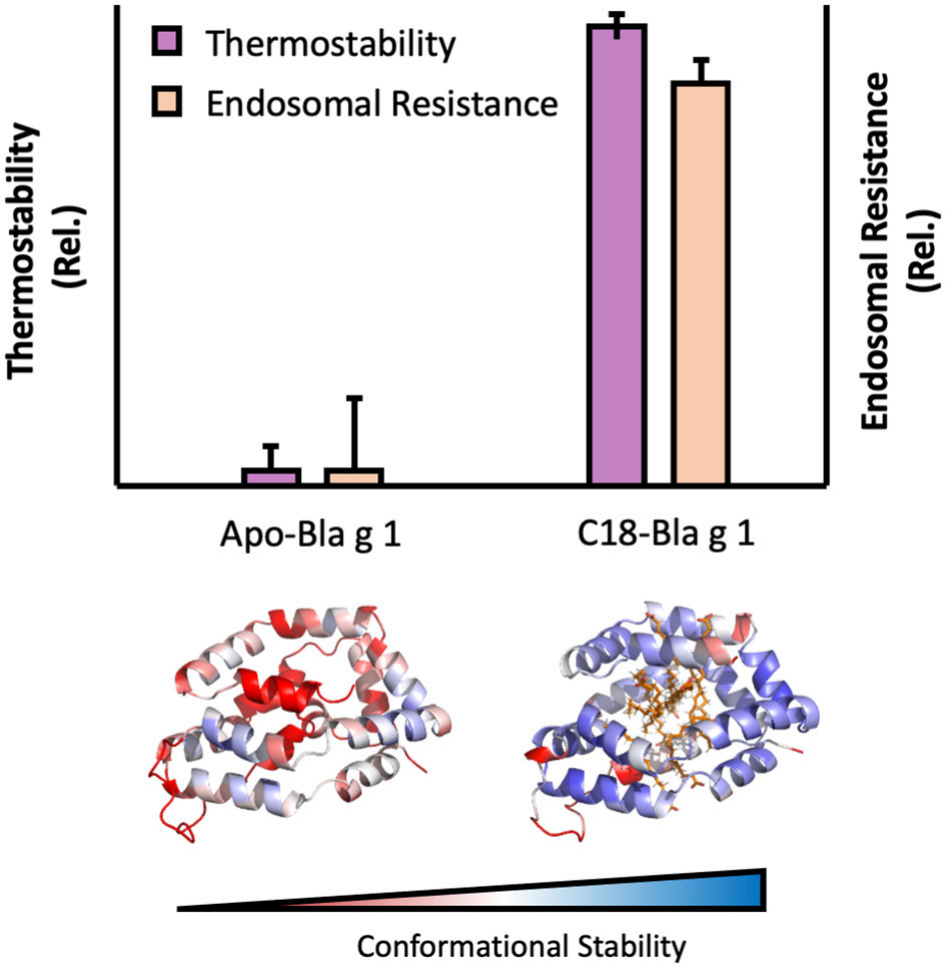
Image showing the influence of ligand binding on Bla g 1 stability. Thermal stability and endosomal processing **(Top)** and backbone (Cα) conformational stability **(Bottom)** of Bla g 1 in the Apo form, or fully loaded with C18 fatty acid ligands. Data replicated from Foo et al. and presented under the Creative Commons CC BY License (122).

Some allergens are also capable of binding specific hydrophobic ligands within a defined cavity or pocket, opening up additional avenues through which endosomal degradation could be altered. This is exemplified by the cockroach allergen Bla g 1. The structure of Bla g 1 encloses an exceptionally large hydrophobic cavity that can accommodate up to 4 phospholipid or 8 fatty-acids, with a mixture of saturated and unsaturated fatty acids representing its natural ligands when purified from its natural allergen source (121, 122). While the unloaded form of Bla g 1 is relatively unstable, binding of its natural fatty-acid ligands or other hydrophobic cargoes into its hydrophobic cavity significantly enhanced conformational stability. This enhancement was strongly dependent on ligand acyl-chain length with 18 carbon (C18) fatty acids yielding maximal stability, and correlated with a decrease in T-cell epitope generation when subjected to Cathepsin proteolysis, providing a direct link between the fold stability and endosomal persistence [Figure 3; (122)]. While Bla g 1 is notable for its large lipid binding capacity (>10% w/w under full stoichiometric binding), similar effects have been observed in less extreme systems. This is best exemplified by the birch allergen Bet v 1, whose structure includes a hydrophobic cavity that can accommodate a variety of ligands, albeit at a much lower stoichiometry of 1–2 (123–125). As with Bla g 1, loading Bet v 1 with phytoprostanes and brassinosteroids from its natural allergen source significantly enhanced fold stability, inhibiting antigen processing and T-cell stimulation (123). Interestingly, one of these Bet v 1 ligands (PPE_1_) was also found to covalently inhibit Cathepsin S, providing an additional mechanism through which hydrophobic ligands can influence the stability, processing, and immunogenicity of allergic proteins (123).

While the ΔG of ligand binding generally enhances the thermodynamic stability of the resulting complex (126), key exceptions exist. One illustrative example can be found among plant Lipid Transfer Proteins (LTP). While binding of their namesake ligands enhanced the thermostability and proteolytic resistance of LTP's from lentils and grapes (127, 128), the reverse effect was observed for their counterparts from wheat (129). The increased susceptibility of the latter was attributed to the displacement of aromatic residues from the hydrophobic core upon ligand binding, resulting in the increased exposure of chymotryptic cut sites and highlighting the need to consider the effects of ligand binding on a case-by-case basis, even when comparing potential allergens within the same protein family.

## Additional Considerations: Biological Activity

Based on the aforementioned studies, we developed a robust model through which the biophysical properties of allergens act to enhance sensitization by increasing the likelihood of exposure and altering the rate of endosomal processing and antigen presentation. This framework also allows us to account for the seemingly anomalous behavior of allergens such as Bet v 1, Bla g 1, and Phl p 4 (29). Even with this mechanistic understanding there are still several proteins which do not follow this trend, highlighting alternative pathways through which antigens can stimulate an allergic response through their biological activities rather than innate biophysical properties. For instance, the mite protein Der p 13 is markedly less stable than its non-allergic counterparts, severely compromising its ability to resist endosomal degradation (28). However, it is still able to induce a Th2 response (130). Biophysical studies identify Der p 13 as a fatty-acid binding protein (FABP) (130). In this capacity it is able to stimulate the membrane-bound pattern-recognition receptor (PRR) TLR2 via the delivery of fatty acid ligands, stimulating production of the pro-inflammatory cytokine IL-8 (130). Der p 13 sensitization was also dependent on Serum Amyloid A (SAA), a soluble lipoprotein which at the time had no known immunological function (131). Subsequent studies identified SAA as a novel, soluble PRR which stimulates the release of IL-33 upon binding of FABP ligands, providing an additional mechanism through which Der p 13 can promote allergic sensitization independent of endosomal degradation and MHCII presentation (131). IL-33 is a pro-inflammatory Danger Associated Molecular Pattern (DAMP) or alarmin that is usually released in response to cellular damage (132). Other lipid-binding allergens such as Bla g 1 and Bet v 1 could ellicit a similar effect through destabilizing the plasma membrane of epithelial cells. In the case of the former, this is mediated by the delivery of unsaturated fatty acids into biological membranes while the latter is able to insert into a lipid bilayer, presumably disrupting lipid organization and packing (114, 133). In this capacity, the lipid-binding abilities of Bet v 1 and Bla g 1 play a dual role in stabilizing their respective ligands while simultaneously contributing to their pro-inflammatory biological functions. These examples highlight the multifaceted nature of allergic sensitization, along with the need to consider not only the biophysical properties of allergenic proteins, but also their interactions with other environmental co-adjuvants or the host's own biological systems. A survey of over 700 allergens indicated that a significant portion (>7%) could be classified as lipid-binding proteins, with some reviews claiming that half of the major allergens share this trait (5, 134). This suggests that the mechanisms outlined above are generally applicable across a wide range of antigenic proteins.

The Radauer study also found that hydrolases, particularly proteases, were equally over-represented among allergenic proteins suggesting that this functionality might also contribute to the sensitization process (5, 135). One mechanism through which this could occur is via the cleavage of proteinase-activated receptor 2 (PAR2), activation of which results in the release of pro-inflammatory cytokines such as IL-8 and IL-33. Indeed, both purified allergens and whole allergen extracts from cockroaches, dust mites and mold were able to stimulate a PAR2 in a proteolytic activity-dependent manner, resulting in a pro-inflammatory response (136–139). It should be noted that proteolytic cleavage of the IL-33 precursor is required to generate the mature, immunologically active cytokine (140). This reaction is normally carried out by inflammatory proteases from activated immune cells. However, this process could be mimicked by an alarming range of environmental and food allergens including both serine (Der p 3, Der p 6) and cysteine (Der p 1) proteases from house dust mites (141–143). Finally, the proteolytic activity of allergens such as Der p 1, Per a 10 (cockroach), and Act d 1 (kiwifruit) can allow them to enhance epithelial barrier permeability through the direct proteolysis of tight junctions and other extracellular structures, facilitating exposure while further triggering alarmin production (144–148). In the case of Der p 1, this proteolytic activity has been shown to enhance the allergenicity of an albumin “bystander” antigen, raising the interesting possibility that biologically-active allergens could act as adjuvants for other components of the exposome. These examples provide ample evidence that biological activity can complement the lackluster stability and/or abundance of allergic proteins such as Der p 1 or Bet v 1 to yield a pro-inflammatory immune response, while the diverse mechanisms through which this enhancement occurs further highlights the multifaceted nature of allergic sensitization.

## Concluding Statements

**Figure 4:**
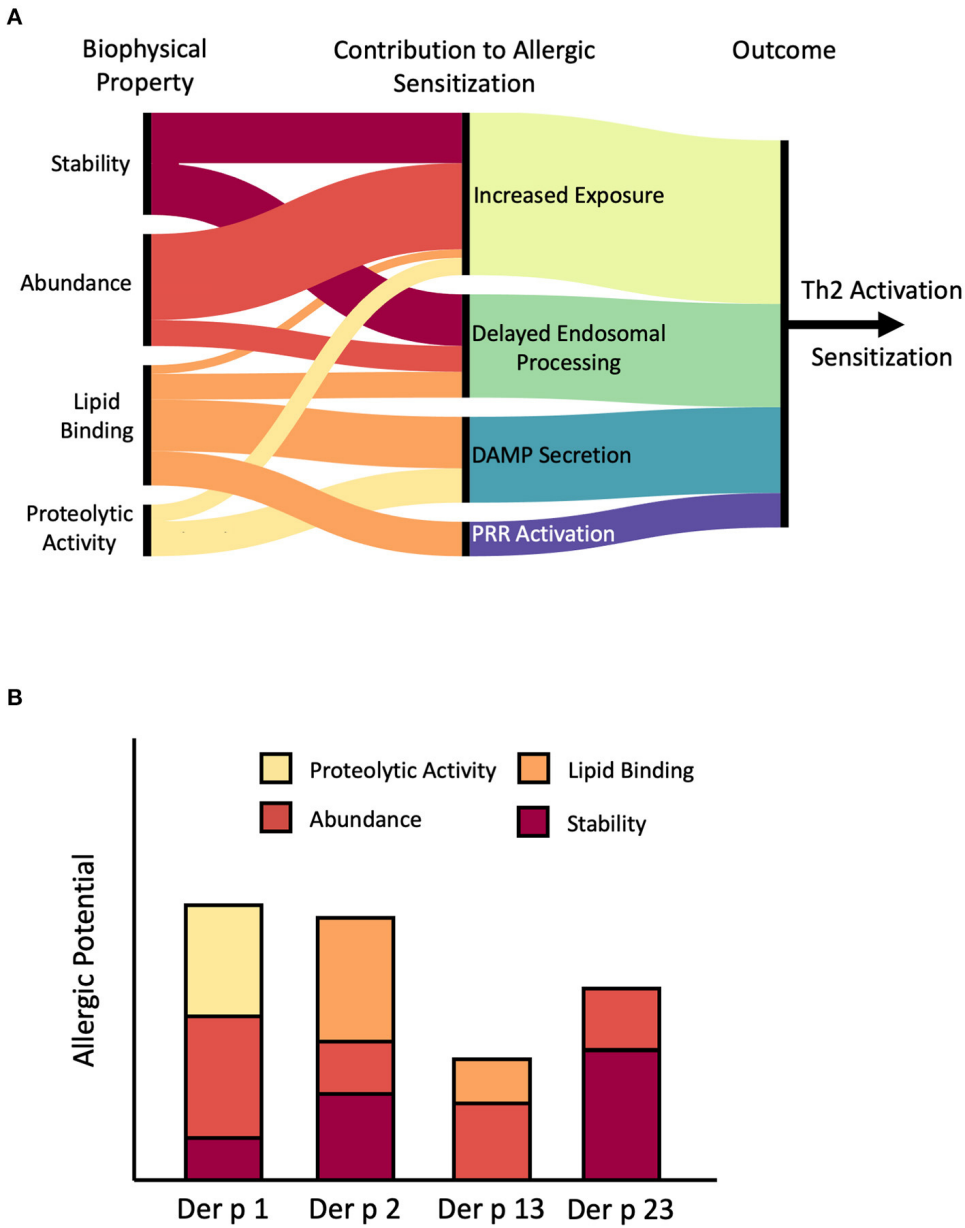
Role of stability, abundance, and biological activity in allergic sensitization. **(A)** Artistic rendition summarizing potential pathways through which stability, abundance, and biological activity can promote allergic sensitization by facilitating exposure to the immune system, altering the kinetics of endosomal processing, triggering the release of danger-associated molecular patterns (DAMP) such as IL-33 and IL-8, or direct stimulation of Pattern-Recognition Receptors (PRR) such as TLR2. Connecting lines depict likely connections. For example, stability may contribute to both increased exposure and delayed endosomal processing. On the other hand, we have found little evidence that proteolytic activity contributes to delayed endosomal processing, hence there is not a connection drawn. Note that the relative contribution of these individual factors can differ between specific allergens, as shown for several example mite allergens in **(B)**. Der p 1 is a major allergen from mites primarily because of proteolytic activity and high abundance while Der p 2 is an abundant lipid-binding allergen. In contrast, Der p 13 is a minor allergen with low stability and moderate abundance and lipid binding capability, while Der p 23 has average abundance, but exceptional stability. Figure generated using RAWGraphs (149).

It is well-established that both abundance and stability contribute to the sensitization process. However, their role is not always as straightforward as one would intuitively expect. While exposure is no doubt a pre-requisite for sensitization, the timing and duration of exposure, rather than its absolute intensity, appear to be the major determinants of allergenicity. Turning to stability, we see a similar relationship, with the kinetics of endosomal processing and MHCII loading representing key steps in the sensitization process. These findings suggest that the correlation between abundance, stability, and allergenicity is not a linear relationship as has been previously thought (18, 19, 92). Instead, there are defined ranges for both parameters within which a protein is most likely to become allergenic. The innate biological activity of many allergens can potentially influence both of these factors, while simultaneously promoting allergic sensitization through other mechanisms such as the release of DAMPS or direct stimulation of PRR's, highlighting the multifaceted nature of allergic sensitization (Figure 4).

The non-linear relationship between stability and allergenicity (Figure 2) raises the possibility for novel immunotherapeutic strategies centered around hypoallergenic variants of naturally occurring allergens with altered stability. For example, stabilized N-terminal truncations of the mite allergen Blo t 5 have been shown to enhance IL-10 production and suppress IL-4, skewing the immune system toward a modified Th2 response (98). Building upon this approach, a hybrid Blo t 5/Blo t 21 molecule has been developed as a potential allergy therapeutic (150). Here, the authors were able to retain the same endosomal degradation patterns while reducing IgE binding to the intact protein (98, 150). The enhanced stability of the hybrid molecule ensured that these resulting endosomal fragments yielded a protective response against their wild-type counterparts. Antigenic Bet v 1 peptide fragments and stabilized Bet v 1 trimers have also been shown to be safe and effective immunotherapeutic candidates (151–153). The latter has been shown to form large aggregates, potentially hindering antigen processing while the former mimics the epitope generation kinetics of a destabilized Bet v 1 variant, suggesting that immunotherapeutic strategies can exploit the bimodal model shown in Figure 2 in both directions. In more extreme examples, whole allergens or antigenic fragments have been conjugated onto stable protein scaffolds. The resulting fusion proteins were able to stimulate the release of tolerogenic cytokines such as IL-10 and IFN-γ, stimulating the production of blocking antibodies (154, 155). Due to the bimodal relationship between stability and allergenicity, the effect of stabilizing/destabilizing mutations is heavily dependent on the biophysical properties of the initial wild-type allergen. This is embodied by the protective effects observed in both stabilized and destabilized Bet v 1 variants described in Figure 2 (88, 156), underscoring the need to carefully consider the nuanced role of stability in allergic sensitization when designing immunotherapeutic candidates.

In addition to enhancing protein stability, hydrophobic ligands are able to directly promote sensitization through a variety of pathways, as summarized in Figure 4. While this additional activity further complicates efforts to develop hypoallergenic variants of Bet v 1 and other lipid-binding allergens, it also opens up additional avenues of development. For instance, Bet v 1 without ligands is deficient in Th2 stimulation, providing a convenient avenue for the development of immunotherapeutic compounds (157). Similarly, the sensitization potential of Ber e 1 and Pru p 3 were also highly dependent on the presence of lipid ligands (158, 159). Given the prevalence of lipid binding among allergens (5), removal and/or replacement of endogenous ligands could provide a simple and convenient approach to generate hypoallergenic compounds for immunotherapeutic applications.

**Figure 5:**
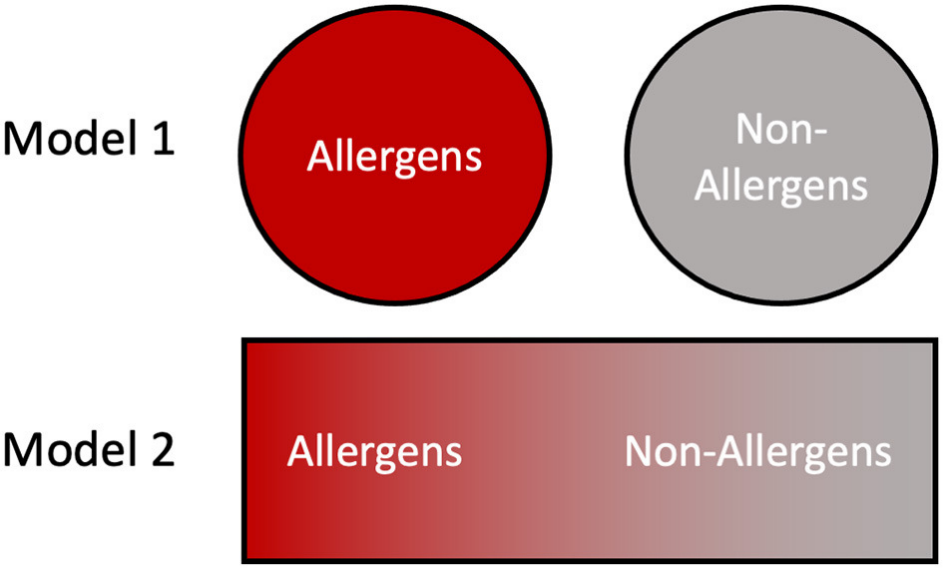
Contrasting views of allergens and non-allergens. In model 1, the division between allergens and non-allergens is binary and absolute, with proteins falling into one of the two categories based on their primary structure features. In model 2 the allergenicity of a protein is dictated by numerous intrinsic and environmental factors such as stability and abundance, resulting in a continuum distribution.

Finally, for this topical edition of Frontiers in Allergy, “What makes an Allergen, an Allergen?”, we present two different models regarding the relationship between allergens and their non-allergen counterparts, which are applicable in different circumstances [Figure 5; (160)]. For example, a clinical allergologist typically thinks of discrete allergens and their use in diagnosing the sensitizing organism, for prescribing appropriate avoidance, and possible therapeutic treatments (161). Similarly, cross-reactivity risks can be assessed using bioinformatics tools and immunological assays against known allergen sequences; clearly these are valuable medical models. However, these strategies assume a binary approach with proteins being classified as either allergens or non-allergens (Figure 5, model 1). When addressing the question, “What makes an allergen, an allergen?”, the data in this paper favors a more probabilistic interpretation in which multiple factors including stability and abundance contribute to allergic sensitization (160). This gives rise to a continuum model (Figure 5, model 2) where, many proteins can become allergens or vice versa depending on the context of presentation and the individual biophysical properties.

## Author Contributions

AF and GM wrote the paper. Both authors contributed to the article and approved the submitted version.

## Funding

This review was supported by the Intramural Research Program of the NIH, National Institute of Environmental Health Sciences, Z01-ES102906 (GM).

## Conflict of Interest

The authors declare that the research was conducted in the absence of any commercial or financial relationships that could be construed as a potential conflict of interest.

## Publisher's Note

All claims expressed in this article are solely those of the authors and do not necessarily represent those of their affiliated organizations, or those of the publisher, the editors and the reviewers. Any product that may be evaluated in this article, or claim that may be made by its manufacturer, is not guaranteed or endorsed by the publisher.
